# Supplementary material for: Identification of photoperiod-induced specific miRNAs in the adrenal glands of Sunite sheep (Ovis aries)
Source: Front Vet Sci. 2022 Jul 22;9:888207. doi: 10.3389/fvets.2022.888207 (PMC9354845; doi:10.3389/fvets.2022.888207)
Supplement: Supplementary file 1 [file Data_Sheet_1.ZIP › Supplementary file/miRDeep2 file.zip!/miRDeep2 file/Know_Structure/SP42A3/oar-mir-150.pdf]

miRBase precursor	: oar-mir-150
Total read count	: 812
oar-miR-150 read count	: 812
remaining reads	: 0

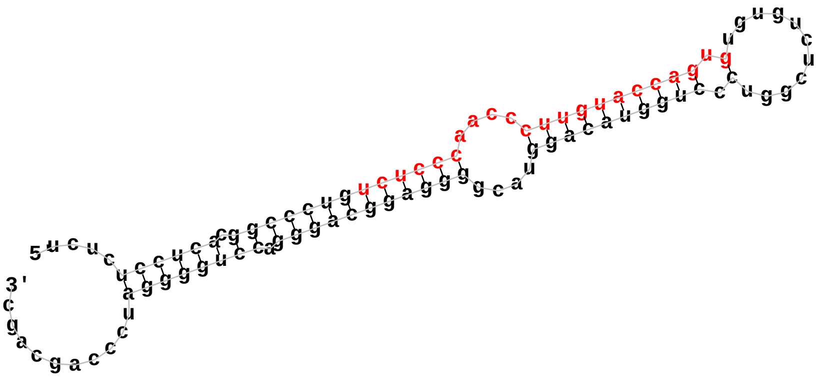

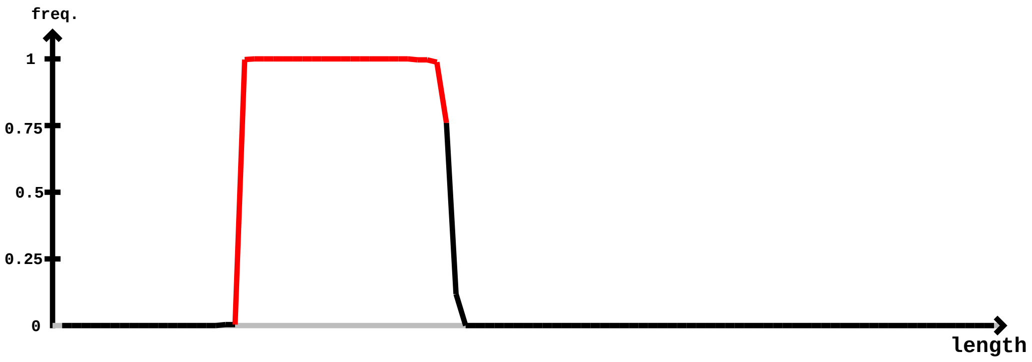


```
                           oar-miR-150
5'- ucucuccucacggcccugucucccaacccuuguaccagugugugucucggucccugguacagguacggggaggcagggaccuggggaucccagcagc   -3'    exp
    ....(((((.((((((((((((((....((((((((((.(...........).))))))))))....))))))))))).)))))))).........    reads      mm          sample
    ................ugucucccaacccuuguaccagu..........................................................    3          0           seq
    ..................ucucccaacccuuguacc.............................................................    3          0           seq
    ..................ucucccaacccuuguaccag...........................................................    7          0           seq
    ..................ucucccaacccuuguaccagu..........................................................    182        0           seq
    ..................ucucccaacccuuguaccagug.........................................................    520        0           seq
    ..................ucucccaacccuuguaccagugu........................................................    95         0           seq
    ...................cucccaacccuuguaccagug.........................................................    1          0           seq
    ...................cucccaacccuuguaccagugu........................................................    1          0           seq
```
